# Changes of renal histopathology and the role of Nrf2/HO-1 in asphyxial cardiac arrest model in rats

**DOI:** 10.1590/ACB360607

**Published:** 2021-07-19

**Authors:** Ali Jawad, Yeo-Jin Yoo, Jae Chol Yoon, Weishun Tian, Md Sadikul Islam, Eui-Yong Lee, Ha-Young Shin, So Eun Kim, Dongchoon Ahn, Byung-Yong Park, Hyun-Jin Tae, In-Shik Kim

**Affiliations:** 1 Fellow Master degree. College of Veterinary Medicine and Bio-safety Research Institute – Jeonbuk National University – Iksan, South Korea.; 2 Fellow PhD degree. College of Veterinary Medicine and Bio-safety Research Institute – Jeonbuk National University – Iksan, South Korea.; 3 Full Professor. Department of Emergency Medicine – Research Institute of Clinical Medicine and Biomedical Research Institute – Jeonbuk National University Hospital – Jeonju, South Korea.; 4 Fellow PhD degree. College of Veterinary Medicine and Bio–safety Research Institute – Jeonbuk National University – Iksan, South Korea.; 5 Fellow Master degree. College of Veterinary Medicine and Bio–safety Research Institute – Jeonbuk National University – Iksan, South Korea.; 6 PhD. Department of Emergency Medicine – Research Institute of Clinical Medicine and Biomedical Research Institute – Jeonbuk National University Hospital – Jeonju, South Korea.; 7 Full Professor. College of Veterinary Medicine and Bio-safety Research Institute – Jeonbuk National University – Iksan, South Korea.; 8 Associate Professor. College of Veterinary Medicine and Bio-safety Research Institute – Jeonbuk National University – Iksan, South Korea.

**Keywords:** Heart Arrest, NF-E2-Related Factor 2, Heme Oxygenase-1, Rats

## Abstract

**Purpose:**

To investigate the role of Nrf2/HO-1 in renal histopathological ailments time-dependently in asphyxial cardiac arrest (CA) rat model.

**Methods:**

Eighty-eight Sprague Dawley male rats were divided into five groups of eight rats each. Asphyxial CA was induced in all the experimental rats except for the sham group. The rats were sacrificed at 6 hours, 12 hours, one day and two days post-CA. Serum blood urea nitrogen (BUN), creatinine (Crtn) and malondialdehyde from the renal tissues were evaluated. Hematoxylin and eosin and periodic acid-Schiff staining were done to evaluate the renal histopathological changes in the renal cortex. Furthermore, Nrf2/HO-1 immunohistochemistry (ihc) and western blot analysis were performed after CA.

**Results:**

The survival rate of rats decreased in a time-dependent manner: 66.6% at 6 hours, 50% at 12 hours, 38.1% in one day, and 25.8% in two days. BUN and serum Crtn markedly increased in CA-operated groups. Histopathological ailments of the renal cortical tissues increased significantly from 6 hours until two days post-CA. Furthermore, Nrf2/HO-1 expression level significantly increased at 6 hours, 12 hours, and one day.

**Conclusions:**

The survival rate decreased time-dependently, and Nrf/HO-1 expression increased from 6 hours with the peak times at 12 hours, and one day post-CA.

## Introduction

Cardiac arrest (CA), also known as circulatory arrest or cardiopulmonary arrest, is a sudden cessation of blood flow to the body due to ineffective heart pumping^1^. Most of CA investigations were conducted to improve the rate of spontaneous circulation (ROSC) over the past half-century, and significant development has been achieved. In contrast, prognosis remains poor even though ROSC can increase with immediate resuscitation^2^,^3^. Post-cardiac arrest syndrome (PCAS), a unique physiological process, can be attributed to the low survival rate of patients after ROSC^1^. The early phase PCAS accounts for a 4 to 33%-survival rate in patients, depending on the survival chain and considering it as the main factor of low survival rate following ROSC^3^,^4^.

Most likely, research work was mainly focused on brain and myocardial injury and dysfunction after CA^4^. The kidney is an important organ in PCAS. Previous findings demonstrated that, following ROSC in patients with PCAS, the dysfunctionality of multiple organs is quite common and related to the low survival rate^5^. However, there is a paucity of information about histopathology and renal ischemia-induced oxidative stress after CA. Furthermore, there is an ambiguous relationship between renal damage and survival rate in PCAS.

The oxidative stress is one of the most important pathways that contribute to the pathogenesis of renal ischemia-reperfusion injury (RI/RI)^6^. Reactive oxygen species (ROS) are generated by an acute oxidative stress response in which blood flow is interrupted to the kidney and its reperfusion subsequently. As a result, ROS overproduction causes lipid peroxidation, mutation of DNA, induced apoptotic with necrotic cascades, that provokes cellular death in different ways^7^,^8^. Nuclear factor-erythroid 2 (Nrf2), an inducible transcription factor, is involved in the regulation of multiple cellular antioxidant systems and limit oxidative stress during ischemia-reperfusion (I/R) induced renal damage^9^. Nrf2 degradation is initiated by the Keap1 (Kelch-like ECH-associated protein-1)-dependent pathway during normal conditions. With the activation of this pathway, disruption of Keap1-Nrf2 binding occurs, as well as transactivation of antioxidant response element(ARE)-driven genes in the nucleus occur, including superoxide dismutase (SOD), glutathione peroxidase (GSH-Px) and Heme oxygenase-1 (HO-1)^10^. In RI/RI pathophysiology, Nrf2 has been regarded as the hub of defense against oxidative stress^9^.

The oxidative stress and Nrf2/HO-1 pathway played a vital role in the I/R injury mechanism. Thus, this signaling pathway was well investigated in the RI/RI studies. However, there is a lack of study on the oxidative stress-induced kidneys via the Nrf2/HO-1 pathway after the CA rat model. The oxidative stress response in the kidney after ROSC might be related to the survival rate in the early and late phase PCAS. Then, we used the asphyxial CA model in rats and determined the survival rate of rats during the post-resuscitation phase. Furthermore, we studied kidneys, histopathological, and pathophysiological ailments induced by renal ischemiaand investigated Nrf2 and HO-1 levels time-dependently after ROSC by immunohistochemistry and western blot.

## Methods

### Experimental animals and groups

All the experimental procedures of this study were approved to Jeonbuk National University-Jbnu (no 2019-005)based on procedures of ethics and scientific care by the institutional animal care and committee at Jeonbuk National University.

Sprague Dawley (SD) rats (270-330 g) were supplied by the Experimental Animal Center of Jeonbuk National University (Iksan campus, South Korea). Eighty-eight rats were used in the experiment, in which 80 rats underwent asphyxial CA surgery and eight were used as the sham group, i.e., did not undergo CA surgery. However, the survival rate was very low. Therefore, 40 rats were used and equally distributed in five groups. Male Sprague Dawley (7 weeks, rats) were divided into sham group(n = 8), and CA-operated rats (n = 80). CA-operated rats were sacrificed at 6 hours (n = 8), 12 hours (n = 8), one day (n =8), and two days (n = 8), following ROSC.

### Induction of cardiac arrest and cardiopulmonary resuscitation

Induction of CA and cardiopulmonary resuscitation (CPR) was done as reported by the published protocol^11^. A rodent ventilator (Harvard Apparatus, United States) was used for anesthesia, and body temperature (37 ± 0.5^o^C)was maintained by heating pads. Peripheral oxygen saturation (SpO_2_) and electrocardiogram (ECG) data were checked regularly in the experiment. The right femoral vein was cannulated for intravenous injection, and the left femoral artery was cannulated for mean arterial pressure (MAP) measurement.

**Figure 1:**
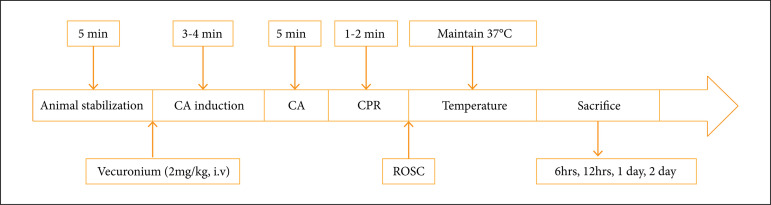
Schematic illustration of the asphyxial cardiac arrest (CA) model with the measurements obtained at animal stabilization (baseline), induction of CA, cardiopulmonary resuscitation (CPR) administration, rate of spontaneous circulation (ROSC) and sacrifice time.

Vecuronium bromide (2 mg/kg) was administered intravenously, and mechanical ventilation was stopped for asphyxial CA induction. After 3-4 min of the stabilization period, when the MAP reached below 25 mmHg, then CA occurred. The bolus of epinephrine injection (0.005 mg/kg) and sodium bicarbonate (1 mg/kg) were administered, after 5 min of CA. Mechanical chest compression was done at300/min with 100% oxygen supply, and when the rats became thermodynamically stable, then they were sacrificed at the specific time points (Fig. 1).

### Measurement of serum blood urea nitrogen and creatinine

Rats were anesthetized with 30% urethane, and 3-5 mL blood was collected from inferior vena cava. Thereafter, it was centrifuged to 4,000 rpm for 15 minutes, and serum was obtained for the determination of blood urea nitrogen (BUN) and creatinine (Crtn) with Automatic Analyzer 7020 (Hitachi, Japan).

### Measurement of malondialdehyde

Malondialdehyde (MDA) concentration in the renal tissues was measured according to the commercial kit instructions. In short, homogenization and centrifugation of renal tissues were done at 4ºC for 10 minutes at10,000 rpm, and its supernatant was kept for experimental analysis at -80^o^C. Optical density of MDA was measured at 535 nm by a tunable *versus* max microplate reader (Cayman Chemical, Ann Arbor, MI, United States).

### Hematoxylin and eosin and periodic acid-Schiff staining

Kidneys were extracted, fixed with 10% neutral buffered formalin, embedded in paraffin, and sectioned (5 µm). Hematoxylin and eosin (H&E) was performed for histopathology^4^. Periodic acid-Schiff (PAS) staining was done to check the glomerular basement membrane. Renal cortical sections were imaged at fixed H&E **×**400 and PAS **×**1,000 magnifications using a Leica DM 2500 microscope (Leica Microsystems, Wetzlar, Germany). Ten fields were analyzed on each histological slide, and one slide was prepared from each rat. Two experienced renal pathologists assessed histopathological changes via quantitative tubulointerstitial injury measurement, based on counting the apoptotic and necrotic cell numbers, loss of tubular brush border, tubular dilatation, cast formation, neutrophil infiltration, and the examination of glomeruli basement membrane thickness in a double-blinded fashion. The scoring was done on the basis of damage:

- 0 = none;
- 1 = 0-10%;
- 2 = 11-25%;
- 3 = 26-45%;
- 4 = 46-75%;
- 5 = 76-100%^12^.

### Immunohistochemistry

Nrf2/HO-1 immunohistochemistry was performed according to our published protocol^13^. In brief, deparaffinization and dehydration of the paraffin sections were done in xylene and ethanol. Antigen retrieval was done with citrate buffer, and 3% hydrogen peroxide was used for the inactivation of endogenous peroxidase activity. Goat serum was used for blocking and incubating the tissue with anti-rabbit polyvalent Nrf2 (Novus bio, catalogue#BP1-32822) and HO-1 (Abcam, catalogue#ab13243)] with antibody dilution (1:500). Subsequently, sections were incubated with the biotinylated secondary antibody (dilution 1:250) and vectastain ABC reagent at room temperature for 1 hour. Diaminobenzidine (DAB) was used for the sections in the dark until the development of brown color. After counterstain,the sections were dehydrated and cleaned in ethanol and xylene and then mounted on a glass slide. Leica DM 2500 microscope (Leica Microsystems, Wetzlar, Germany) was used to image the sections at fixed ×400 magnification. From each group, ten areas were captured. Image-J threshold analysis software (ij152-win-Java8) was used to measure the relative optical density percentage (ROD%).

### Western blot analysis

To investigate the protein level of Nrf2/HO-1 in the renal cortical tissues, western blot analysis was carried out by our previously published method^4^. In short, renal cortical tissues were lysed using lysis buffer, then bicinchoninic acid (BCA) protein assay protein kit was used for the evaluation of the total protein concentration of lysate tissues. A proportionate amount of protein was isolated and passed to a nitrocellulose membrane using 12% sodium dodecyl sulfate-polyacrylamide gel electrophoresis (SDS-PAGE). The incubation of the membrane was done with 5% bovine serum albumin (BSA) for 2 hours and then for overnight in the primary antibody. After washing and incubating with secondary antibodies, enhanced chemiluminescence (ECL) detection kit was used for the detection of bands, and the images of bands were captured by a LAS-400 image system (GE Healthcare, Little Chalfont, United Kingdom). ?-actin was used as the reference antibody.

### Statistical analysis

Data were expressed as the standard error mean (SEM) using GraphPad Prism 5.0. Survival data were analyzed by using Kaplan-Meier statistics and log-rank tests. One-way analysis of variance (ANOVA) was used to compare the groups followed by Bonferroni’s multiple comparison tests. Shapiro-Wilk test was performed to evaluate the normality of the samples, and p < 0.05was considered statistically significant for all the analysis.

## Results

### Physiological variables

There were insignificant (p < 0.05) changes between the CA-operated groups and the sham group for baseline characteristics (Table 1). MAP and SpO2 with isoelectric ECG were used to confirm CA, but changes were observed in the ECG, MAP, and SpO2 as expected according to the protocol. The survival rate of rats was 66.6% at 6 hours, 50% at 12 hours, 38.1% at one day, and 25.8% at two days after ROSC. At the baseline and after ROSC, body temperature, body weight and heart rate did not change. Moreover, the room temperature was kept normal during the experiment.

**Table 1:** Physiologic variables and survival rate.

Variables	Baseline	Cardiac arrest	6-Hour post cardiac arrest	12-Hour post cardiac arrest	1-Day post cardiac arrest	2-Day post cardiac arrest
Survival rate	100%	-	66.6%	50%	38.1%	25.8%
Body weight (g)	275 ± 16	-	279 ± 37	277 ± 43	281 ± 55	279 ± 45
Mean arterial pressure (mmHg)	119 ± 15	-	116 ± 18	109 ± 34	117 ± 31	113 ± 35
Asphyxial time to CA (s)	-	-	192 ± 33	189 ± 41	186 ± 46	195 ± 23
Cardiopulmonary resuscitation time (s)	-	-	1.4 ± 0.4	1.6 ± 0.2	1.6 ± 0.4	1.5 ± 0.8
Heart rate (beat/min)	331 ± 13	-	329 ± 54	340 ± 55	348 ± 52	329 ± 56
Body temperature (ºC)	36.8 ± 0.2	35.5 ± 0.61	36.8 ± 0.26	36.8 ± 0.51	36.9 ± 0.21	37 ± 0.1
Room temperature (ºC)	-	24.7 ± 0.51	25.3 ± 0.81	24.9 ± 0.49	25.1 ± 0.81	25.3 ± 0.33

### Assessment of the renal function

**Figure 2:**
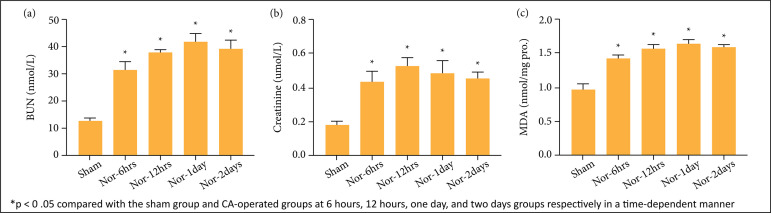
Blood urea nitrogen (BUN) **(a)**, creatinine (Crtn) **(b)**, and malondialdehyde (MDA) **(c)** were significantly higher in the cardiac arrest (CA)-operated groups compared to the sham group. Data are expressed as mean ± standard error means.

Serum BUN and Crtn were assessed to evaluate the renal function of experimental animals. BUN and Crtn increased significantly (p < 0.05) at 6 hours, 12 hours, one day, and two days. Furthermore, the peak point of BUN and Crtn was at 12 hours and one day post-CA group (Fig. 2 a-b).

### Malondialdehyde levels in renal tissues

MDA concentration in the kidney was measured using the instructions of commercial kit (Cayman Chemical, Ann Arbor, MI, United States) (Fig. 2c). Following 5 minutes of ischemia and reperfusion caused increase of MDA concentration in the CA-operated groups compared with those in the sham group (p < 0.05).

### Renal histopathology

**Figure 3:**
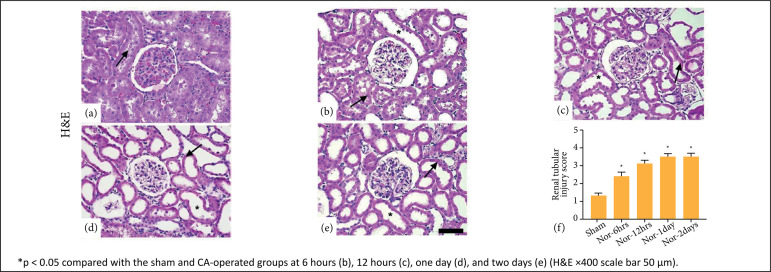
Hematoxylin and eosin (H&E) staining of the sham group **(a)** has shown no tubular injury. In cardiac arrest (CA)-operated groups [**b**-6h, **c**-12h, **d**-1day, **e**-2days], the tubular injury score is markedly increased at 6 hours **(b)** and maintained until two days **(e)**. Renal tubules showed severe dilatation*, loss of brush border with necrosis (*arrow*). Data are expressed as mean ± standard error means (SEM).

**Figure 4:**
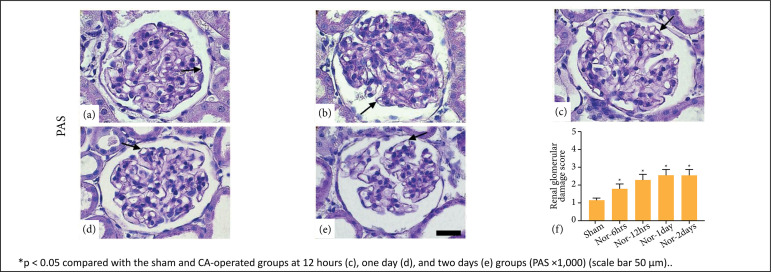
Periodic acid-Schiff (PAS) staining of the sham group **(a)** indicated no damage in the glomerular basement membrane. In cardiac arrest (CA)-operated groups [**b**-6h, **c**-12h, **d**-1day,e-2days], glomerular basement membrane injury score markedly increased at 12 hours **(c)** until two days **(e)**, as shown with the arrow. Data are expressed as mean ± standard error means.

The renal histopathological changes of the CA-operated groups increased significantly post-CA (p<0.05). The histopathology showed loss of the tubular brush border, glomerular capillaries dilatation with less severely inflammatory cells, and acute tubular necrosis. Based on the H&E score, proximal and distal convoluted tubular damages were markedly increased at 6 hours post-CA in the renal cortex area. At 12 hours post-CA, damages were significantly increased and maintained until two days of post-CA (Fig. 3). PAS stain showed that the damage score for the diameter of glomeruli capillaries with little change in the thickness of the glomerular basement membrane was increased significantly at 12 hours, one day, and two days after ROSC as compared to the sham. It also increased after 6 hours post-CA, but not significantly (Fig. 4).

### Immunohistochemical analysis of Nrf2/HO-1

**Figure 5:**
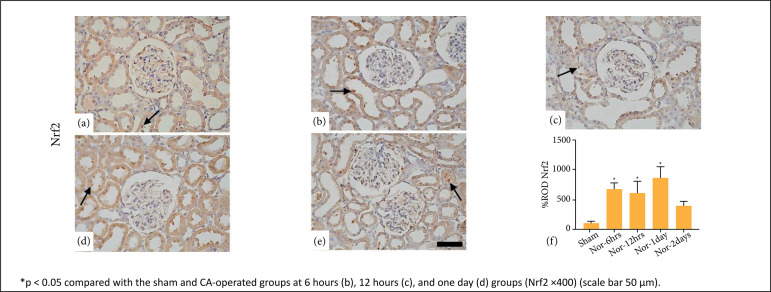
Immunohistochemistry of Nrf2 in the kidney of the sham **(a)** and cardiac arrest (CA)-operated [**b**-6h, **c**-12h, **d**-1day, **e**-2days] groups. Relative optical density (ROD%) of Nrf2 expression is significantly increased at 6 hours **(b)**, 12 hours **(c)**, and one day **(d)** after rate of spontaneous circulation in CA-operated groups as compared to the sham group (*arrow*). Data are expressed as mean ± standard error means.

**Figure 6:**
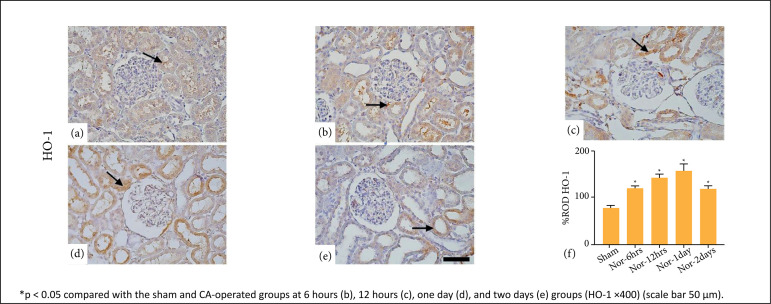
Immunohistochemistry of HO-1 in the kidney of the sham **(a)** and cardiac arrest (CA)-operated [**b**-6h, **c**-12h, **d**-1day, **e**-2days] groups. Relative optical density (ROD%) of HO-1 expression is significantly increased at 6 hours **(b)**, 12 hours **(c)**, one day **(d)**, and two days **(e)** after rate of spontaneous circulation in CA-operated groups compared to the sham (*arrow*). Data are expressed as mean ± standard error means (SEM).

**The expression of Nrf2 and HO-**1 in renal tissues of CA-operated groups was higher (p < 0.05) as compared to the sham group. Few tubular cells were stained in the sham group. However, the other groups showed an increase in the number of tubular cells stained with Nrf2 and HO-1 in a time-dependent manner. Stained cell numbers were mild at 6 hours, moderate at 12 hours, and marked at one day. However, the number of stained cells decreased at two days post-CA (Figs. 5 and 6).

### Western blot analysis

**Figure 7:**
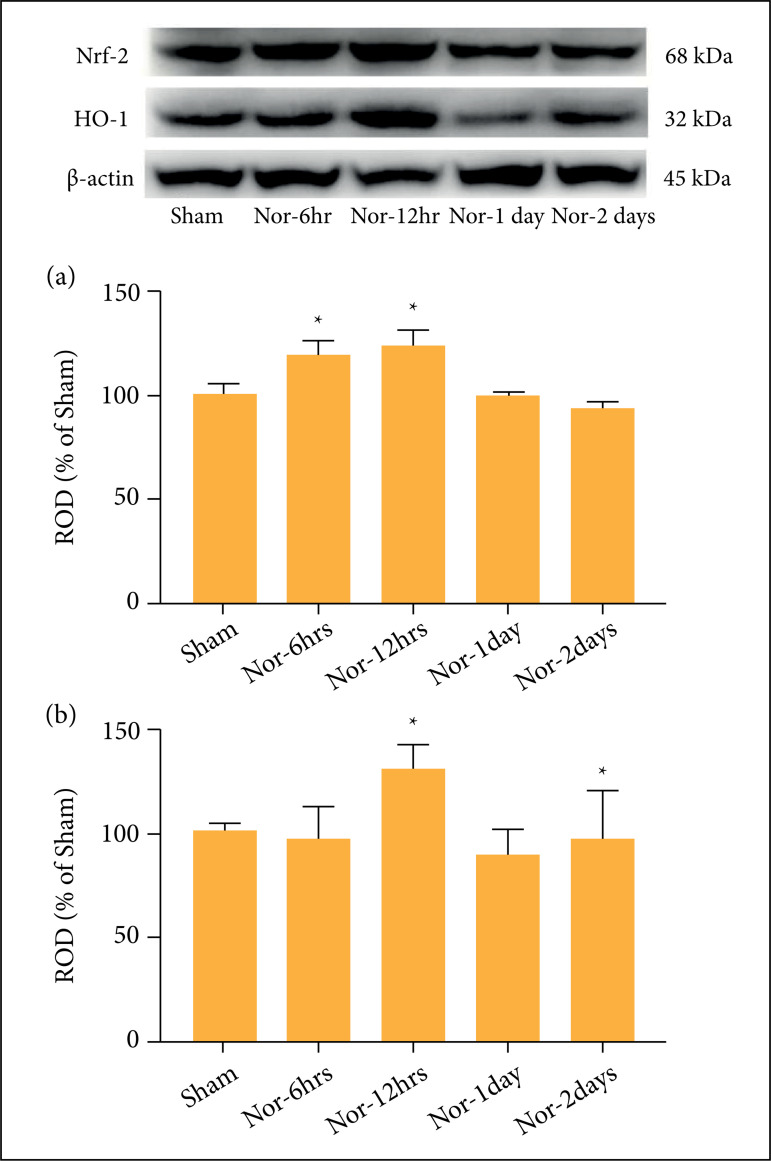
Western blot analysis of Nrf2/HO-1 in the renal cortex of the sham and cardiac arrest (CA)-operated groups. Relative optical density (ROD%) of Nrf2 expression **(a)** was significantly increased at 6 hours and 12 hours, but ROD% of HO-1 expression **(b)** was significantly increased at 12 hours and two days after ROSC, as compared to the sham. All the data are expressed as mean ± standard error means (SEM).

In our study, the western analysis was conducted to evaluate the antioxidative response in the kidney after ROSC (Fig. 7). Western blot analysis indicated that Nrf2/HO-1 was significantly increased in the CA-operated groups compared with the sham group. The relative optical density (ROD%) of Nrf2 expression increased significantly at 6 and 12 hours. The ROD% of HO-1 expression increased in a significant manner at 12 hours and two days post-CA. The peak time of expression for both Nrf2 and HO-1 is 12 hours after ROSC.

## Discussion

In CA patients, the survival rate accounted for 4 to 39% in out-of-hospital CA patients with their CPR done by the ambulance staff, and they survived via admission. The death of more than half of them occurred within the first 24 hours in the hospital^4^,^14^. In this study, the survival rate decreased time-dependently and reached to 25.8% at two days post-CA, what shows similarity with the survival rate of the CA patients. Therefore, our CA model is favorable for CA patients^14^.

After RI/RI, the most common histopathological features included were cast formation, loss of brush border of proximal tubular cells, tubular necrosis, and tubular dilatation and expansion^12^. In the present study, severe dilatation of the renal tissues, loss of the brush border of proximal convoluted tubules, tubular necrosis, and enlarged glomerular capillaries were seen in the histological studies of the kidney that shows consistency with the previous studies of RI/R^12^.

Fu *et al*.^15^ demonstrated that BUN and Crtn significantly increased at one and two days following ROSC in their CA model in rats. In this study, the level of BUN and Crtn increased significantly at 12 hours, one day and two days after CPR following ROSC, which shows consistency with their CA model^15^. Despite the difference of ischemia duration, experimental model and animal, the histopathological and pathophysiological results of this study were similar to RI/RI experiments^6^,^15^. Previous studies demonstrated that reduced number of the glomeruli in pigs and rats were observed following 21 and 30 days after warm renal ischemia of 30 and 60 minutes. In contrast, the present study showed no reduction in the glomeruli number, but the survival rate decreased in a time-dependent manner after CPR. The discrepancy might be related to the experimental technique in which the ischemia duration was 30 and 60 minutes and only the kidney was affected. However, in the present study, asphyxial CA caused the whole-body ischemia-reperfusion injury with only 5 minutes of ischemia-duration^16^,^17^.

Oxidative stress plays a pivotal role in the RI/RI development, which is pathologically induced by the overproduction of ROS and reactive nitrogen species (RNS)^18^. In the reperfusion process, highly electrophilic ROS outburst results in perturbing renal redox balance state, that directly causes the breakdown of DNA, inactivation of protein, renal structural and functional tubular cell damage by extensive membrane lipid peroxidation^19^. In this study, MDA significantly increased in CA-operated groups compared to the sham group and showed similarity with the previous results of RI/RI^6^,^20^. Therefore, the present study suggests that our asphyxial CA involvesRI/RI mechanism resulting in oxidative stress induction and provokes renal damage^20^.

Nrf2, a cap’n’collar (CNC) basic leucine zipper (bZIP) redox-sensitive transcription factor, influencing intrinsic resistance to oxidative stress by the induction and production of ROS-detoxifying enzymes with antioxidants, including thioredoxin (TXN), sulfiredoxin (SRYN), tripeptide glutathione (GSH), can cause the reduction of oxidized protein thiols^21^. Jiang *et al*.^6^ reported that the Nrf2/HO-1 expression increased at one day post-reperfusion in RI/RI rat model. In the present study, Nrf2 expression increased significantly (p < 0.05) at normothermia one day group post-CA, which shows consistency with the result of the RI/RI model. Previous study showed that the maximum expression of Nrf2 in the kidney at 8 hours of reperfusion in the ischemic AKI model in mice^22^.

In this asphyxial CA model in rats, the Nrf2 expression trend is similar to the ischemic AKI model in which Nrf2 expression is at its peak level at 8 hours and one day^22^. Most of the RI/RI researches were conducted at one day after reperfusion to check the expression of Nrf2. However, we checked its expression at 6 hours, 12 hours, one day and two days, respectively. The high level of Nrf2 expression was significantly maintained until seven days in the ischemic preconditioning (IPC) RI/RI model of rats with the attenuation of renal injury^23^. IPC is a short and non-lethal episodic ischemia that protects the kidney against ischemia insults via the upregulation of endogenous protective mechanisms. Therefore, increased Nrf2 expression was significantly maintained in the kidney, and it also attenuated the renal injury and dysfunction in IPC RI/RI until seven days. In contrast, the present study suggests that the Nrf2 expression increased at 6 hours, 12 hours until one day, and decreased at two days and induces severe renal injury andlow survival rate at two days post-CA in the asphyxial CA model^23^.

HO-1 degrades heme into biliverdin and CO and later converts biliverdin into bilirubin, that acts as a powerful antioxidant in several disease models including RI/RI^24^. Previous research suggested that the HO-1 expression vigorously increased at one day, and its elevation was observed until five days after RI/RI in mice^25^. In this study, HO-1 expression increased at 6 hours, 12 hours and one day post-CA in a time-dependent manner after ROSC. At two days post-CA, HO-1 expression decreased compared to the other groups, showing a similar trend of expression as conducted in the RI/RI models^25^. In previous studies, mice were used for RI/RI model with 45 minutes of ischemia duration and to induce renal dysfunction. However, we used rats for the asphyxial CA model with ischemia timing of 5 minutes and whole-body ischemia-reperfusion injury (WBIRI) occurred^25^.

In spite of the difference in the experimental model and animal, our results were consistent with their studies of RI/RI until one day, and then the expression of HO-1 decreased at two days post-CA. The plausible cause is that, after ROSC, when the I/R occurs, the whole body is held accountable for acting as an additional stimulus in AKI. The impairment in multiple organs occurs simultaneously with the I/R injury, in which the detrimental product releasedinto the circulation and aftershock scavenging function. Thus, we considered that the expression of HO-1 decreased at two days post-CA, despite the short ischemia-duration (5 minutes) in our asphyxial CA model in rats.

Nrf2/HO-1 signaling pathway has illustrated multiple cytoprotective roles against apoptosis, inflammatory, and oxidative stress in the kidney^26^. The previous study reported that IPC causes the upregulation of Nrf2/HO-1 expression in the I/R induced kidney injury at one day after reperfusion in mice^27^. In IPC RI/RI experiments, the high expression of Nrf2/HO-1 confers protection against oxidative stress-induced via renal ischemia insults^27^. Thus, the present study indicated the upregulation of Nrf2/HO-1 at one-day post-CA at its peak level. Therefore, we assumed that the Nrf2/HO-1 showed resistance against oxidative stress until one day after ROSC. At two days post-CA, the expression of Nrf2/HO-1 decreased, suggesting the threshold point as theresistance against oxidative stress halted and inducedthe progression of renal injury and the low survival rate in our asphyxial CA model. Thus, we assumed that the expression of Nrf2/HO-1 decreased, the renal injury and dysfunction increased, and the survival rate of rats decreased at two days post-CA.

The present study strengths showed that the Nrf2/HO-1 signaling pathway is putative in renal injury with dysfunction. This pathway plays the role of the important protective factors for the favorable prognosis of survival of death in PCAS after CA. The present study has some shortcomings. In this study, the survival rate suddenly decreased at 6 hours (66.6%). However, patients continue to die, and mortality usually increases as time goes by. In this study, HO-1 ROD% decreased at one day showing the sham group’s pattern. Furthermore, an electron microscope was not used for the evaluation of the glomerular basement membrane in the present study. These are the potential limitations of the present study. Therefore, further study is required to investigate the mechanism of mortality, evaluating the glomerular lesions via the electron microscopy and Nrf2/HO-1 expression level in the asphyxial CA rat model.

## Conclusion

The histopathological score, renal function assessment markers, and MDA content with severe renal injury increased and were maintained until two days post-CA. In short,Nrf2/HO-1 expression increased, and the survival rate decreased in the time-dependent manner post-CA.
